# Resurrection of the genus Aphyllon for New World broomrapes (Orobanche s.l., Orobanchaceae)

**DOI:** 10.3897/phytokeys.75.10473

**Published:** 2016-12-09

**Authors:** Adam C. Schneider

**Affiliations:** 1 Jepson Herbarium and Department of Integrative Biology, 1001 Valley Life Sciences Building, University of California, Berkeley, CA 94720-2465

**Keywords:** Aphyllon, broomrape, Gymnocaulis, Myzorrhiza, Nothaphyllon, nomenclature, Orobanche, Orobanchaceae

## Abstract

Recent phylogenetic studies support a monophyletic clade of New World broomrapes (Orobanche sects. Gymnocaulis and Nothaphyllon) sister to the Old World genus Phelipanche. I place the New World taxa in the genus Aphyllon, propose 21 new combinations, and provide a list of currently accepted taxa.

## Introduction

Phylogenetic analysis of broomrapes and related holoparasites using nuclear DNA have found that the small eastern Mediterranean genus Diphelypaea Nicolson is nested within Orobanche sensu lato (s.l.) as circumscribed by Beck (1890) (Schneeweiss et al. 2004a). Morphological and cytological differences between groups of taxa within Orobanche s.l. have led some botanists to adopt a narrower generic circumscription. In this taxonomic concept, Orobanche sensu stricto is limited to Old World species that lack bracteoles and have a base chromosome number of *x* = 19, a calyx divided to the base, and generally unbranched stems (Holub 1977, 1990). Other Old World broomrapes are treated as Phelipanche Pomel or the monotypic genus Boulardia F.W. Schultz (syn: Orobanche sect. Trionychon Wallr. and Orobanche latisquama (F.W. Schultz) Batt., respectively; Joel 2009; Schneweiss 2013).

Broomrape species native to the New World constitute two well-supported clades that together form a clade sister to Phelipanche (Schneider et al. 2016). Taxonomically, these clades have been recognized as two separate genera Aphyllon (= Orobanche sect. Gymnocaulis Nutt.) and Myzorrhiza Phil. (= Orobanche sect. Nothaphyllon (A. Gray) Heckard) by Holub (1977, 1990) and others (Schneweiss 2013), or more rarely, together as Aphyllon s.l. (Gray 1876). However, neither of these generic taxonomies has been widely adopted among American botanists, in part because of the lack of available names for many taxa. Providing evidence to support the treatment of all New World broomrapes as Aphyllon and a providing list of recognized species (with homotypic synonymns) is the purpose of this paper. New combinations are made where appropriate.

## Methods

In order to compare molecular branch lengths of major clades of Orobanche s.l., a maximum likelihood (ML) phylogram of Aphyllon and related holoparasites was inferred from 3 nuclear DNA loci (ITS, phytochrome A, and phytochrome B). All sequences were downloaded from Genbank, aligned, and concatenated into a supermatrix using SUMAC (Freyman 2015). The ML phylogeny was estimated using RAxML (Stamatakis 2014) with a GTR+Γ nucleotide substitution model and 1000 rapid bootstrapping replicates.

Information about type specimens, basionyms, and synonomy of these new combinations was gathered by examining protologues and images of type specimens using major databases, including Tropicos (http://www.tropicos.org), JSTOR Global Plants (http://plants.jstor.org), and the International Plant Names Index (http://www.ipni.org). Types for all North American taxa and Orobanche weberbaueri Mattf. have been designated by previous authors and are presented here. For three of the four South American taxa, typification would require more careful efforts beyond the scope of this article. No repository is given in the protologue for two syntypes of Orobanche tacnaensis Mattf. (Woitschach 71 and F. J. F. Meyen s.n.). The current existence of these specimens could not be verified, although a photograph of the Woitscach 71 (possibly from a specimen at B) is available at F. No specimens are cited by Rodolfo Phillipi in the protologues of the two taxa that he described.

## Discussion

**Figure 1.**
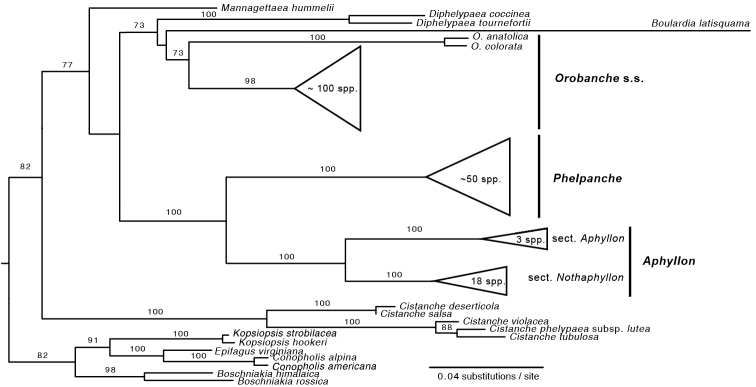
Maximum likelihood (ML) phylogram of Aphyllon and related holoparasite species inferred from 3 nuclear DNA loci (ITS, phytochrome A and phytochrome B). Bootstrap support values >70% are labeled. Due to space constraints, several clades have been collapsed. For a more detailed and thorough study of phylogenetic relationships within Aphyllon, see Schneider et al. (2016).

Molecular phylogenetic analyses have consistently supported a sister-group relationship between two strongly supported two American clades, representing Orobanche sect. Gymnocaulis and Orobanche sect. Nothaphyllon (McNeal et al. 2013; Schneider et al. 2016; Fig. 1). This relationship is supported by biogeography and synapomorphies such as a calyx with five fully developed lobes and a base chromosome number of *x* = 12, with polyploidy in most taxa (Heckard and Chuang 1975; Schneeweiss et al. 2004b). Holub (1977, 1991) has proposed treating the American broomrapes as two genera rather than one, though this was likely due to his erroneous belief based on vegetative morphology that Orobanche sect. Nothaphyllon is most closely related to Phelipanche and that Orobanche sect. Gymnocaulis is allied to Orobanche sect. Orobanche (Holub, 1977).

The genus Aphyllon was described by Mitchell (1769), although it was not until nearly 80 years later that Asa Gray made a combination for Aphyllon uniflorum A. Gray. This species was the only broomrape included in his *Manual of the Botany of the Northern United States* (1848), though in the second edition (1856) Gray recognized two additional species. Gray limited his generic concept for Aphyllon to taxa assignable to Orobanche sect. Gymnocaulis, instead recognizing Orobanche ludoviciana Nutt. in Phelipaea Tourn. ex Desf. However, after a study of the Californian flora, Gray amended his generic concept of Aphyllon to include two sections, Aphyllon and Nothaphyllon, together containing all taxa native to the New World (Gray 1876). Though expanded from Gray’s initial circumscription, it was appropriate given Michell’s original diagnosis of Aphyllon as having a five-toothed calyx (“semiquinquefidum”), a synapomorphy of New World broomrapes. A generation later, Rydberg (1906) proposed elevating Gray’s Aphyllon sect. Nothaphyllon to genus rank on the basis of differences in habit and placentation using the available name Myzorrhiza Phil. However, the broader generic concept of Orobanche used by Beck (1890) has prevailed, particularly among American botanists.

Due to the biogeographical, morphological, cytological, and phylogenetic affinities of the New World broomrapes, I recommend treating them in a single genus, Aphyllon, composed of sections Aphyllon (= Orobanche sect. Gymnocaulis) and Nothaphyllon (= Orobanche sect. Nothaphyllon). Below, I present a key to sections and a list of recognized taxa in Aphyllon, proposing new combinations as necessary. Combinations are made at the most recently treated rank for the taxon in Orobanche, with the exception of Orobanche uniflora subsp. *occidentale* Greene, which is recognized at species rank under the available name Aphyllon purpureum (A. Heller) Holub due to its unique hosts, long molecular branch lengths, and recent discovery of sympatric populations of Aphyllon purpureum and Aphyllon uniflorum in southwestern British Columbia (Schneider et al. 2016). The treatment of Aphyllon sect. Aphyllon should be considered tentative; further taxonomic study is underway which will result in the recognition of several additional taxa.

### Key to sections of Aphyllon

**Table:** 

1	Bracteoles subtending the calyx absent; pedicels much longer than flower (2-8× length); stems subterranean or rising to about ground level	**Aphyllon sect. Aphyllon** (syn.: Orobanche sect. Gymnocaulis)
1’	Bracteoles subtending the calyx 2; pedicels equal to or shorter than flower, occasionally 2× length; stems usually rising above ground level	**Aphyllon sect. Nothaphyllon** (syn.: Orobanche sect. Nothaphyllon)

## Taxonomic treatment

### Aphyllon

Taxon classificationPlantaeLamialesOrobanchaceae

Mitch., Diss. Brevis. Princ. Bot. 43. 1769.

Loxanthes Raf. Neogenyton 3. 1825. [Type: Loxanthes fasciculatus (Nutt.) Raf.]
Anoplanthus Endl., *nom. superfl.*, Gen. Pl. [Endlicher] pt. 10: 727. 1839.
Thalesia Raf. ex Britton, *nom. superfl.*, Mem. Torrey Bot. Club 5: 298. 1894.

#### Type.

Aphyllon uniflorum (L.) Torr. & A. Gray, Manual 290. 1848.

#### Description.

Herb, annual or rarely perennial, achlorophyllous, holoparasitic. Stems fleshy. Leaves reduced to scale-like bracts. Inflorescences terminal racemes, spikes, corymbs, or panicles. Calyx 5-toothed. Corolla sympetalous, bilabiate to regular, tubular and often curved. Style long, stigma crateriform and peltate, or bilamellar. Fruit loculicidal capsules.

About 22 species: 18 in North America, 4 in South America.

### Aphyllon sect. Aphyllon

Taxon classificationPlantaeLamialesOrobanchaceae

Orobanche sect. Gymnocaulis Nutt., Gen. N. Amer. Pl. [Nuttall]. 2: 59. 1818.

#### Description.

Stems subterranean or rising to about ground level. Pedicels long and slender, much longer than flower. Bracteoles subtending the calyx absent.

### Aphyllon fasciculatum

Taxon classificationPlantaeLamialesOrobanchaceae

(Nutt.) Torr & A. Gray, Manual (ed. 2) 281. 1848.

Orobanche fasciculata Nutt., Gen. N. Amer. Pl. 2: 59. 1818.
Phelipaea fasciculata (Nutt.) Spreng., Syst. Veg. [Sprengel] 2: 818. 1825.
Loxanthes fasciculatus (Nutt.) Raf., Neogenyt. 3. 1825.
Anoplon fasciculatum (Nutt.) G. Don., Gen. Hist. 4: 633. 1838.
Anoplanthus fasciculatus (Nutt.) Walp., Repert. Bot. Syst. 3: 480. 1844.
Thalesia fasciculata (Nutt.) Britton, Mem. Torrey Bot. Club 5: 298. 1894.

#### Type.

USA: “Missouri”, ca. 1811, *Nuttal s.n.*, (holotype, PH).

### Aphyllon purpureum

Taxon classificationPlantaeLamialesOrobanchaceae

(A. Heller) Holub, Preslia 70: 100. 1998.

Thalesia purpurea A. Heller, Bull. Torrey Bot. Club 24: 313. 1896.
Orobanche porphyrantha Beck, Pflanzenr. 96[IV,261]: 49. 1930.
Orobanche uniflora var. purpurea (A. Heller) Achey, Bull. Torrey Bot. Club 60: 445. 1933.

#### Type.

USA: Idaho: Nez Perce Co.: near mouth of the Potlatch, 20 May 1896, *Heller 3099.* (no holotype designated; isotypes, CAS, DAO, K, MIN, MO, MSC, NDG, PH, US).

### Aphyllon uniflorum

Taxon classificationPlantaeLamialesOrobanchaceae

(L.) Torr & A. Gray, Manual (Gray) 290. 1848

Orobanche uniflora L., Sp. Pl. 2: 633. 1753.
Anoplanthus uniflorus (L.) Endl., Gen. Pl. [Endlicher] 727. 1839.
Thalesia uniflora (L.) Britton, *Mem. Torrey Bot. Club* 5: 298. 1894.

#### Type locality.

USA: Virginia (lectotype, *Clayton 387*, BM).

### Aphyllon sect. Nothaphyllon

Taxon classificationPlantaeLamialesOrobanchaceae

A. Gray, Bot. California [W.H. Brewer] 1: 584. 1876

Myzorrhiza Phil., Linnea 29: 36. 1858. [Type: Myzorrhiza chilensis Phil.]
Orobanche sect. Myzorrhiza Beck, Bibliotheca Botanica 4(19): 78. 1890.
Orobanche sect. Nothaphyllon (A. Gray) Heckard, Madroño 22: 41. 1973.

#### Type.

Aphyllon californicum (Cham. & Schltdl.) A. Gray, lectotype designated by Heckard, Madroño 22: 41. 1973.

#### Description.

Stems clearly rising above ground. Pedicels equal to or shorter than flower. Bracteoles subtending the calyx 1 or 2.

### Aphyllon arizonicum

Taxon classificationPlantaeLamialesOrobanchaceae

(L.T. Collins) A.C. Schneid. comb. nov.

urn:lsid:ipni.org:names:77158997-1

Orobanche arizonica L.T. Collins, *Phytoneuron* 2015–48: 16, f. 1, 2, 4, 5, 6A, 7. 2015.

#### Type.

USA: Arizona, Coconino Co.: near Tuba City, 1539 m, 27 September 1935, *Kearney & Peebles 12867* (holotype, ARIZ; isotype, US).

### Aphyllon californicum

Taxon classificationPlantaeLamialesOrobanchaceae

(Cham. & Schltdl.) A. Gray, Bot. California 1: 584. 1876.

Orobanche californica Cham. & Schltdl., *Linnea* 3: 134–136. 1828.
Phelypaea californica (Cham. & Schltdl.) G. Don, *Gen. Hist.* 4: 632. 1838.
Myzorrhiza californica (Cham. & Schltdl.) Rydb., *Bull. Torrey Bot. Club* 36: 696. 1909.

#### Type.

USA: California: Near Port of San Francisco, Aug 1816, Chamisso s.n (holotype, LE).

### Aphyllon californicum subsp. condensum

Taxon classificationPlantaeLamialesOrobanchaceae

(Heckard) A.C. Schneid. comb. nov.

urn:lsid:ipni.org:names:77159010-1

Orobanche californica subsp. condensa Heckard, *Madroño* 22: 59–60, f. 1I-L, 5. 1973.

#### Type.

USA: California: San Luis Obispo Co.: Yaro Creek, 25 May 1955, *Bacigalupi, Ferris & Robbins 5242* (holotype, JEPS; isotypes, NY, RSA, US, WTU).

### Aphyllon californicum subsp. feudgei

Taxon classificationPlantaeLamialesOrobanchaceae

(Munz) A.C. Schneid. comb. nov.

urn:lsid:ipni.org:names:77159005-1

Orobanche grayana var. feudgei Munz, *Bull. Torrey Bot. Club* 57: 616–617, pl. 38, f. 8. 1930.
Orobanche californica subsp. feudgei (Munz) Heckard, *Madroño* 22: 62. 1973.

#### Type.

USA: California: San Bernardino Co.: Baldwin Lake, 2 June 1924, *Munz 8177* (holotype, POM).

### Aphyllon californicum subsp. grande

Taxon classificationPlantaeLamialesOrobanchaceae

(Heckard) A.C. Schneid. comb. nov.

urn:lsid:ipni.org:names:77159011-1

Orobanche californica subsp. grandis Heckard, *Madroño* 22: 60–62, f. 1P-R, 3A, 4E, 5. 1973.

#### Type.

USA: California: Santa Barbara Co.: dunes at Surf, 22 July 1954, *H. M. Pollard* (holotype, UC; isotype, CAS).

### Aphyllon californicum subsp. grayanum

Taxon classificationPlantaeLamialesOrobanchaceae

(Beck) A.C. Schneid. comb. nov.

urn:lsid:ipni.org:names:77159006-1

Orobanche grayana Beck, *Biblioth. Bot.* 4: 79. 1890.
Myzorrhiza grayana (Beck) Rydb., *Bull. Torrey Bot. Club* 36: 695. 1909.
Orobanche californica subsp. grayana (Beck) Heckard, *Madroño* 22: 54. 1973.

#### Type.

USA: Oregon: banks of the Columbia River, 1825, *Douglas s.n.* (lectotype, K).

### Aphyllon californicum subsp. jepsonii

Taxon classificationPlantaeLamialesOrobanchaceae

(Munz) A.C. Schneid. comb. nov.

urn:lsid:ipni.org:names:77159007-1

Orobanche grayana var. jepsonii Munz, *Bull. Torrey Bot. Club* 57: 617, pl. 38, f. 10. 1930.
Orobanche californica subsp. jepsonii (Munz) Heckard, *Madroño* 22: 57. 1973.

#### Type.

USA: California: Colusa Co.: Princeton, October 1905, *H. P. Chandler s.n.* (holotype: POM, isotype: UC).

### Aphyllon chilense

Taxon classificationPlantaeLamialesOrobanchaceae

(Phil.) A.C. Schneid. comb. nov.

urn:lsid:ipni.org:names:77158998-1

Myzorrhiza chilensis Phil., *Linnea* 29: 36–37. 1857.
Orobanche chilensis (Phil.) Beck, *Biblioth. Bot.* 4: 82–83. 1890.

### Aphyllon cooperi

Taxon classificationPlantaeLamialesOrobanchaceae

A. Gray, Proc. Amer. Acad. Arts 20: 307. 1885.

Orobanche ludoviciana var. cooperi (A. Gray) Beck, Biblioth. Bot. 4(Heft 19): 81. 1890.
Orobanche cooperi (A. Gray) A. Heller, Cat. N. Amer. Pl. 7. 1898.
Myzorrhiza cooperi (A. Gray) Rydb. *Bull. Torrey Bot. Club* 36: 695. 1909.

#### Type locality.

USA: Arizona: Fort Mojave (lectotype designated by Munz, Bull. Torrey Bot. Club 57: 620-21, *Cooper* s.n. in 1860-61, GH).

### Aphyllon cooperi subsp. latilobum

Taxon classificationPlantaeLamialesOrobanchaceae

(Munz) A.C. Schneid. comb. nov.

urn:lsid:ipni.org:names:77159008-1

Orobanche ludoviciana var. latiloba Munz, *Bull. Torrey Bot. Club* 57: 621–622, pl. 39, f. 18. 1930.
Orobanche cooperi subsp. latiloba (Munz) L.T. Collins, *Phytoneuron* 2015–48: 15. 2015.

#### Type.

USA: California: Riverside Co.: Colorado Desert, 22 April 1922, *Munz & Keck 4960* (holotype: POM, isotype, US).

### Aphyllon cooperi subsp. palmeri

Taxon classificationPlantaeLamialesOrobanchaceae

(Munz) A.C. Schneid. comb. nov.

urn:lsid:ipni.org:names:77159009-1

Orobanche multicaulis var. palmeri Munz, *Bull. Torrey Bot. Club* 57: 613, pl. 38, f. 2. 1930.
Orobanche cooperi subsp. palmeri (Munz) L.T. Collins, *Phytoneuron* 2015–48: 16. 2015.

#### Type.

Mexico, Durango, April-November 1896, *Palmer 7* (holotype: GH, isotypes, MO, UC).

### Aphyllon corymbosum

Taxon classificationPlantaeLamialesOrobanchaceae

(Rydb.) A.C. Schneid. comb. nov.

urn:lsid:ipni.org:names:77158999-1

Myzorrhiza corymbosa Rydb., *Bull Torrey Bot. Club* 36: 696. 1909.
Orobanche corymbosa (Rydb.) Ferris, *Contr. Dudley Herb.* 5: 99. 1958.

#### Type.

USA: Reynold’s Creek, 2 July 1892, *Isabel Mulford s.n.* (holotype, NY; isotype, MO).

### Aphyllon corymbosum subsp. mutabile

Taxon classificationPlantaeLamialesOrobanchaceae

(Heckard) A.C. Schneid. comb. nov.

urn:lsid:ipni.org:names:77159012-1

Orobanche corymbosa subsp. mutabilis Heckard, *Canad. J. Bot.* 56: 187–188. 1978.

#### Type.

USA: Washington: Grant Co.: O’Sullivan Dam, 11 July 1950, *S. W. Harris 97* (holotype, WS).

### Aphyllon dugesii

Taxon classificationPlantaeLamialesOrobanchaceae

S. Watson, Proc. Amer. Acad. Arts 18: 132. 1883.

Orobanche dugesii (S. Watson) Munz, Bull. Torrey Bot. Club 57: 613, t. 38, f. 3. 1931.

#### Type.

Mexico: Gueanajatao, *Dugès s.n.* (holotype, GH).

### Aphyllon ludovicianum

Taxon classificationPlantaeLamialesOrobanchaceae

(Nutt) A. Gray. Bot. California [W.H.Brewer] 1. 585.

Orobanche ludoviciana Nutt. Gen. N. Amer. Pl. 2: 58–59. 1818.
Phelypaea ludoviciana (Nutt) Walp. Repert. Bot. Syst. 3: 461. 1844.
Myzorrhiza ludoviciana (Nutt) Rydb. Fl. S.E. U.S 1338. 1903.

#### Type.

USA: Fort Mandan, 1810-1811, *Nuttall s.n.* (holotype, PH).

### Aphyllon multiflorum

Taxon classificationPlantaeLamialesOrobanchaceae

(Nutt) A. Gray. Bot. California [W.H.Brewer] 1. 585.

Orobanche multiflora Nutt., J. Acad. Nat. Sci. Philadelphia, ser. 2 1: 179. 1848.

#### Type.

USA: Rio Grande, 1845, *Gambel s.n.* (neotype designated by White & Holmes, Sida 19: 623, USA: Texas: Jim Wells Co., 19 April 1944, *Lundell & Lundell* 12809, LL; isoneotype, LL).

### Aphyllon parishii

Taxon classificationPlantaeLamialesOrobanchaceae

(Jeps.) A.C. Schneid. comb. nov.

urn:lsid:ipni.org:names:77159001-1

Orobanche californica var. parishii Jeps. *Man. Fl. Pl. Calif.* 952. 1925.
Orobanche parishii (Jeps.) Heckard. *Madroño* 22: 66. 1973.

#### Type.

USA: California: San Bernardino Co.: Bear Valley, 1894, *S. B. Parish s.n.* (holotype, JEPS).

### Aphyllon parishii subsp. brachylobum

Taxon classificationPlantaeLamialesOrobanchaceae

(Heckard) A.C. Schneid. comb. nov.

urn:lsid:ipni.org:names:77159013-1

Orobanche parishii subsp. brachyloba Heckard, *Madroño* 22: 68–70, 2J, 3N, 5. 1973.

#### Type.

USA: California: Ventura Co.: Dutch Harbor, San Nicolas Island, 23 April 1966, *Raven & Thompson 20794* (holotype, JEPS; isotypes, MO, RSA, SBBG).

### Aphyllon pinorum

Taxon classificationPlantaeLamialesOrobanchaceae

(Geyer ex Hook.) A. Gray, Bot. California 1: 585. 1876.

Orobanche pinorum Geyer ex Hook., *Hooker’s J. Bot. Kew Gard.* 3:297–298. 1851.

#### Type.

USA: Idaho/Washington border, *Geyer 445* (holotype, K).

### Aphyllon riparium

Taxon classificationPlantaeLamialesOrobanchaceae

(L.T. Collins) A.C. Schneid. comb. nov.

urn:lsid:ipni.org:names:77159002-1

Orobanche riparia L.T. Collins, *J. Bot. Res. Inst. Texas* 3: 7–10, f. 1A-B, 2. 2009.

#### Type.

USA: Indiana, Gibson Co.: Griffin, 16 August 1931, *Deam 50941* (holotype, IND; isotypes, A, F, GH, IND, MINN, WIS).

### Aphyllon robbinsii

Taxon classificationPlantaeLamialesOrobanchaceae

(Heckard ex Colwell & Yatsk.) A.C. Schneid. comb. nov.

urn:lsid:ipni.org:names:77159015-1

Orobanche robbinsii Heckard ex Colwell & Yatsk., *Phytoneuron* 2016-58: 2. 2016.

#### Type.

USA: California: San Francisco Co.: Lands End, 13 August 1956, *Robbins 3707* (holotype, JEPS; isotypes, CAS, GH, NY).

### Aphyllon tacnaense

Taxon classificationPlantaeLamialesOrobanchaceae

(Mattf.) A.C. Schneid. comb. nov.

urn:lsid:ipni.org:names:77159016-1

Orobanche tacnaensis Mattf., *Notizbl. Bot Gart. Berlin-Dahlem* 8: 185–186. 1922.

#### Syntypes.

Peru: Tacna, 1890, *Woitschach 71* (photograph of type: F); Peru: Tacna, 1833, *F. J. F. Meyen s.n.*

### Aphyllon tarapacanum

Taxon classificationPlantaeLamialesOrobanchaceae

(Phil.) A.C. Schneid. comb. nov.

urn:lsid:ipni.org:names:77159003-1

Orobanche tarapacana Phil., *Anales Mus. Nac. Santiago de Chile* 1891: 69. 1891.

### Aphyllon tuberosum

Taxon classificationPlantaeLamialesOrobanchaceae

(A. Gray) A. Gray, Bot. California 1: 585. 1876.

Phelypaea tuberosa A. Gray, *Proc. Amer. Acad. Arts* 7: 371. 1868.
Orobanche bulbosa Beck, *Biblioth. Bot.* 4: 83–84. 1890.

#### Type.

USA: California: Monterey Co: Gavilan Mountains, 1860-1862, *Brewer 743* (holotype, GH; isotype K).

### Aphyllon validum

Taxon classificationPlantaeLamialesOrobanchaceae

(Jeps.) A.C. Schneid. comb. nov.

urn:lsid:ipni.org:names:77159017-1

Orobanche valida Jeps., *Madroño* 1: 255–256. 1929.
Orobanche ludoviciana var. valida (Jeps.) Munz, *Bull. Torrey Bot. Club* 57: 621. 1930.

#### Type.

USA: California: Rock Creek, San Gabriel Mountains, 2 June 1923, *F. W. Peirson 7937* (holotype: JEPS, isotype: RSA).

### Aphyllon validum subsp. howellii

Taxon classificationPlantaeLamialesOrobanchaceae

(Heckard & L.T Collins) A.C. Schneid. comb. nov.

urn:lsid:ipni.org:names:77159018-1

Orobanche valida subsp. howellii Heckard & L.T Collins, *Madroño* 29: 98–100, f. 1A–E. 1982.

#### Type.

USA: California: Mendocino Co.: Impassable Rock, 14 July 1951, *Donald V. Hemphill s.n.* (holotype: UC).

### Aphyllon vallicolum

Taxon classificationPlantaeLamialesOrobanchaceae

(Jeps.) A.C. Schneid. comb. nov.

urn:lsid:ipni.org:names:77159004-1

Orobanche comosa var. vallicola Jeps., *Man. Fl. Pl. Calif.* 952. 1925.
Orobanche vallicola (Jeps.) Heckard, *Madroño* 22: 64. 1973.

#### Type.

USA: California: Santa Clara Co.: Coyote, 14 October 1914, *W. L. Jepson 6196* (holotype: JEPS, isotypes: GH, MO).

### Aphyllon weberbaueri

Taxon classificationPlantaeLamialesOrobanchaceae

(Mattf.) A.C. Schneid. comb. nov.

urn:lsid:ipni.org:names:77159019-1

Orobanche weberbaueri Mattf., *Notizbl. Bot Gart. Berlin-Dahlem* 8: 185. 1922.

#### Type.

Peru: Camaná: Areuipa, Hafen Chala, 26 November 1915, *A. Weberbauer 7185* (isotypes: GH, US).

## Supplementary Material

XML Treatment for Aphyllon

XML Treatment for Aphyllon sect. Aphyllon

XML Treatment for Aphyllon fasciculatum

XML Treatment for Aphyllon purpureum

XML Treatment for Aphyllon uniflorum

XML Treatment for Aphyllon sect. Nothaphyllon

XML Treatment for Aphyllon arizonicum

XML Treatment for Aphyllon californicum

XML Treatment for Aphyllon californicum subsp. condensum

XML Treatment for Aphyllon californicum subsp. feudgei

XML Treatment for Aphyllon californicum subsp. grande

XML Treatment for Aphyllon californicum subsp. grayanum

XML Treatment for Aphyllon californicum subsp. jepsonii

XML Treatment for Aphyllon chilense

XML Treatment for Aphyllon cooperi

XML Treatment for Aphyllon cooperi subsp. latilobum

XML Treatment for Aphyllon cooperi subsp. palmeri

XML Treatment for Aphyllon corymbosum

XML Treatment for Aphyllon corymbosum subsp. mutabile

XML Treatment for Aphyllon dugesii

XML Treatment for Aphyllon ludovicianum

XML Treatment for Aphyllon multiflorum

XML Treatment for Aphyllon parishii

XML Treatment for Aphyllon parishii subsp. brachylobum

XML Treatment for Aphyllon pinorum

XML Treatment for Aphyllon riparium

XML Treatment for Aphyllon robbinsii

XML Treatment for Aphyllon tacnaense

XML Treatment for Aphyllon tarapacanum

XML Treatment for Aphyllon tuberosum

XML Treatment for Aphyllon validum

XML Treatment for Aphyllon validum subsp. howellii

XML Treatment for Aphyllon vallicolum

XML Treatment for Aphyllon weberbaueri
